# Supplementary figures and images for: Human Gut Phageome Analysis Uncovers Thousands of Highly Modular Endolysins
Source: Microbiologyopen. 2026 Jun 21;15(3):e70344. doi: 10.1002/mbo3.70344 (PMC13284279; doi:10.1002/mbo3.70344)

# Unique Domain

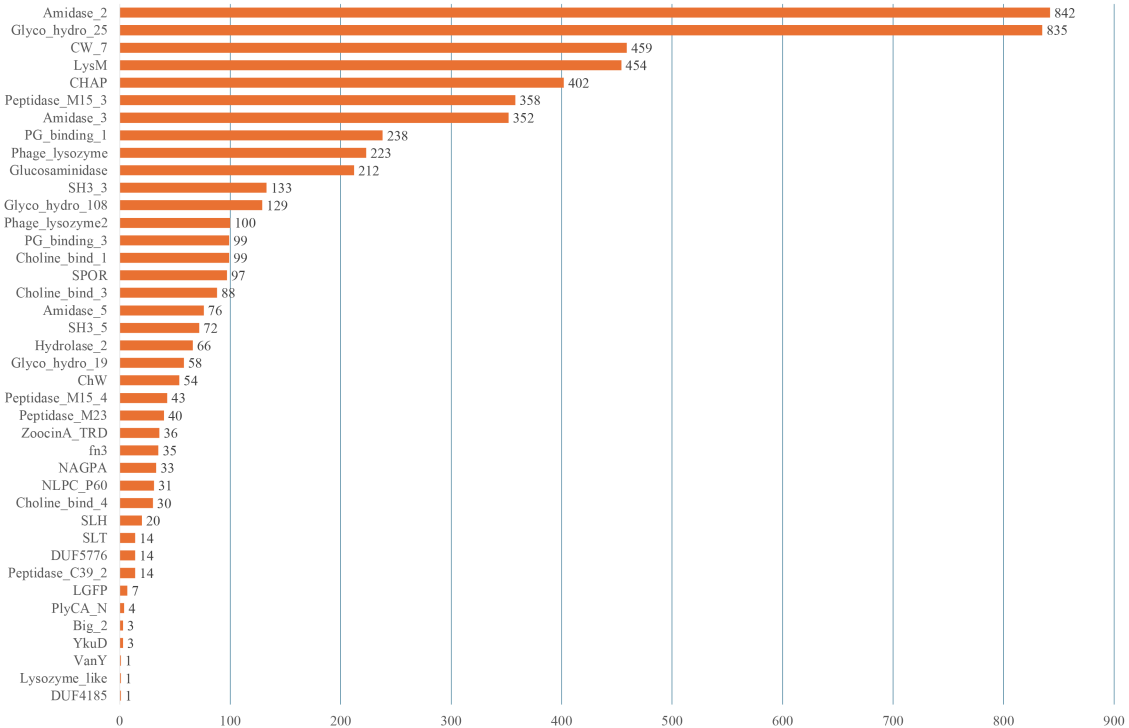

Supplement: Supplementary file 1 — Figure S1: Total distribution of endolysin domains in the human gut phageome. [file MBO3-15-e70344-s006.pdf]
