# Supplementary material for: Human Gut Phageome Analysis Uncovers Thousands of Highly Modular Endolysins
Source: Microbiologyopen. 2026 Jun 21;15(3):e70344. doi: 10.1002/mbo3.70344 (PMC13284279; doi:10.1002/mbo3.70344)
Supplement: Supplementary file 2 — Figure S2: Radial maximum‐likelihood phylogenetic tree of endolysin proteins of human gut phageome. Concentric rings (inner to outer) denote phamily, phage family, phage lifestyle, phage genome orientation, and host bacterial phylum. Branch lengths represent substitutions per site. The figure illustrates the broad evolutionary and ecological diversity of gut phage‐encoded endolysins. [file MBO3-15-e70344-s004.pdf]

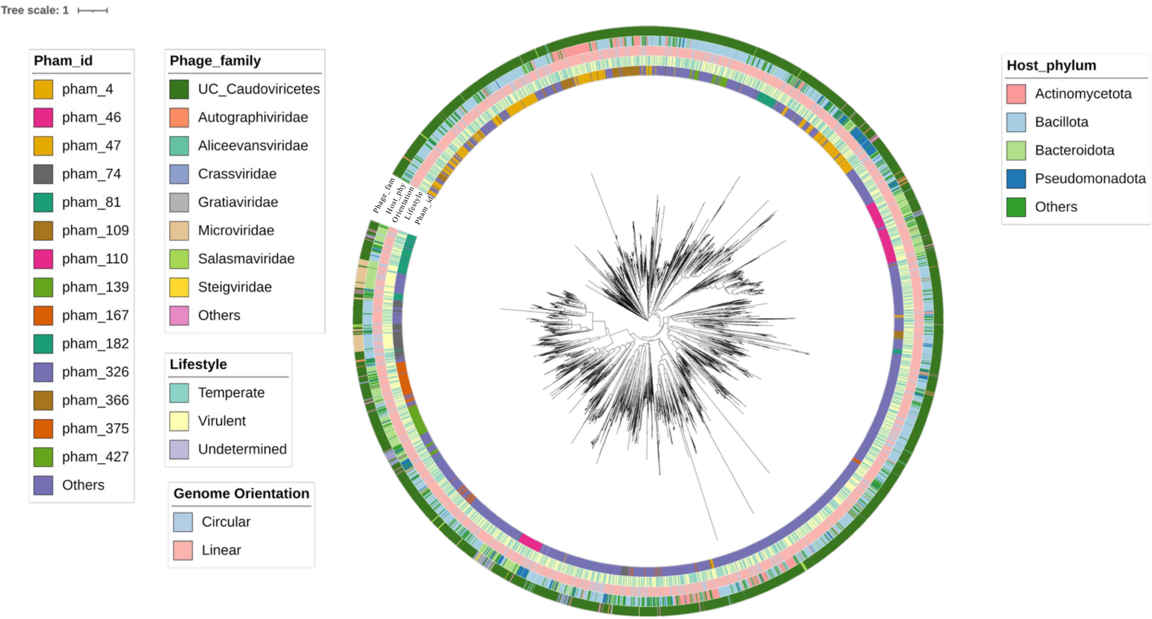

Tree scale: 1
Phage_fam
Host_phy
Orientation
Lifestyle
Pham_id
Pham_id
pham_4
pham_46
pham_47
pham_74
pham_81
pham_109
pham_110
pham_139
pham_167
pham_182
pham_326
pham_366
pham_375
pham_427
Others
Phage_family
UC_Caudoviricetes
Autographiviridae
Aliceevansviridae
Crassviridae
Gratiaviridae
Microviridae
Salasmaviridae
Steigviridae
Others
Lifestyle
Temperate
Virulent
Undetermined
Genome Orientation
Circular
Linear
Host_phylum
Actinomycetota
Bacillota
Bacteroidota
Pseudomonadota
Others
